# An Artificial Intelligence Approach Toward Food Spoilage Detection and Analysis

**DOI:** 10.3389/fpubh.2021.816226

**Published:** 2022-01-12

**Authors:** Ekta Sonwani, Urvashi Bansal, Roobaea Alroobaea, Abdullah M. Baqasah, Mustapha Hedabou

**Affiliations:** ^1^Department of Computer Science and Engineering, Dr. B. R. Ambedkar National Institute of Technology, Jalandhar, India; ^2^Department Computer Science, College of Computers and Information Technology, Taif University, Taif, Saudi Arabia; ^3^Department of Information Technology, College of Computers and Information Technology, Taif University, Taif, Saudi Arabia; ^4^School of Computer Science, Mohammed VI Polytechnic University, Ben Guerir, Morocco

**Keywords:** machine learning for health, smart system, food spoilage detection, food spoilage prevention, sensors, IoMT

## Abstract

Aiming to increase the shelf life of food, researchers are moving toward new methodologies to maintain the quality of food as food grains are susceptible to spoilage due to precipitation, humidity, temperature, and a variety of other influences. As a result, efficient food spoilage tracking schemes are required to sustain food quality levels. We have designed a prototype to track food quality and to manage storage systems at home. Initially, we have employed a Convolutional Neural Network (CNN) model to detect the type of fruit and veggies. Then the proposed system monitors the gas emission level, humidity level, and temperature of fruits and veggies by using sensors and actuators to check the food spoilage level. This would additionally control the environment and avoid food spoilage wherever possible. Additionally, the food spoilage level is informed to the customer by an alert message sent to their registered mobile numbers based on the freshness and condition of the food. The model employed proved to have an accuracy rate of 95%. Finally, the experiment is successful in increasing the shelf life of some categories of food by 2 days.

## 1. Introduction

Food wastage has been a topic of concern in recent years, and studies are being conducted to identify innovative ways to reduce it. It has been described as a major concern in the long-term sustainability of food production, demand, and food supply chains. Since meals are the essential source of diet for all living beings, the quality and security of meals have always been in high demand. The Internet of Things (IoT) links anything, everywhere, and at any time (1–4) and by incorporating the IoT into the Food Supply Chain (FSC) management, it is possible to improve food shelf life by measuring and monitoring the state of the food and exchanging data to and from customers. Currently, the whole use of IoT technologies in FSC is only in its early stages, with a long way to go (5, 6). To avoid food wastage, food sanitation and safety are of paramount importance. The consistency of meals should be regulated. As a result, wastage can be reduced by installing quality control systems in grocery stores. It will further help in controlling diseases (7). These types of quality control systems keep an eye on the environmental conditions that may maintain the food quality. Previously, atmospheric effects were monitored by procedures such as refrigeration, vacuum storage, and so on. Food pollution can occur during the manufacturing process, although it is most often caused by inefficient food handling activities due to unsuitable environmental conditions during food transportation and storage.

### 1.1. Food Spoilage Process

**Figure 1:**
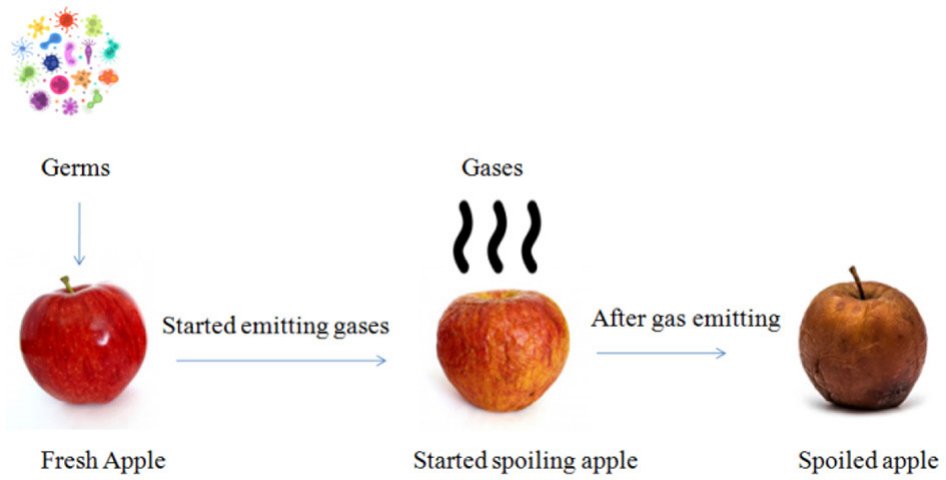
Food spoilage process.

Food spoilage occurs when a food product becomes unfit for use by the customer. The factors of such a process are a result of a variety of external factors, including the type of food item, how it is packaged and processed. Every year, one-third of the world's melded are lost which is provided for human consumption due to meal wastage (8, 9). In Figure 1, it could be visualized that once the germs start attacking fresh apples, gas emission takes place and after few hours, the apple is spoiled completely.

Bacteria, virus, protozoa, and fungi are factors of food spoilage. These factors can create harmful results for consumers, but we can apply prevention techniques to them to save the life and quality of food. Usually, bacteria can not cause food poisoning and most of the microorganisms that cause food borne illness are odorless and tasteless, otherwise owing to mycotoxins and microbial wastes. Therefore, eating spoiled food is never recommended. Two pathogenic bacteria *Clostridium perfringens* and *Bacillus cereus* can spoil the food.

### 1.2. Key Component of Food

There is a very important key component i.e., seed. If we want healthy fruits and veggies, that seed must be strong and healthy. The fruits and veggies completely depend on the seeds. There are two types of seeds:

- **Hybrid seeds:** Cross-pollinated plants produce hybrid seeds in cultivation and gardens. Hybrids are selected for their ability to enhance the characteristics of the resulting plants, such as yield, uniformity, color, and disease resistance.
- **Non-hybrid seeds:** These seeds are called heirloom seeds or free pollinated seeds. Non-hybrid seeds originate from naturally pollinated trees. Any of these cultivars have been around for millennia.

The hybrid seeds give hybrid fruits and vegetables and non-hybrid seeds give non-hybrid fruits and veggies. The lifetime of hybrid fruits and veggies is less than the non-hybrid fruits and veggies. But everyone cannot identify which fruit or vegetable is hybrid or not; that is why everybody needs to know the knowledge of their routine work.

Food poisoning can be caused by a variety of factors, including humidity and temperature fluctuations. As a result, it is important to provide a measurement device that can measure humidity and temperature differences during food preparation and transportation (10). Currently, nearly everyone is influenced by the foods they consume daily, not only because of junk food, but also because of canned vegetables and other food items eaten in daily life, which lack consistency because their oxygen, temperature, and moisture content differ from time to time. In the smart house, devices are installed which have the capacity to diagnose wastage of food and then alert caregivers. The study and execution of repetitive measurements, which are aimed at detecting improvements, do not guarantee the nutritional content of the food (11). The details gathered by tracking and monitoring should be reviewed and properly submitted to the administrator for purposes of policy research, pattern forecasting, program assessment, and planning.

The expanded use of smart phones, connected devices, and controllers in food production and other domains has wreaked havoc across the globe. The advantages of IoT technology are rapidly being tapped by the food industry and other sectors (12–14). It accesses *via* broadband (modem) and begins scanning input from the wired up to sensors, the heat, and humidity sensor until properly mounted and turned on. The temperature and moisture controller sensor is a wireless sensor with an active moisture controller sensor. We have employed this idea in the proposed scheme.

Again, the primary function of the proposed monitoring and control systems is to keep track of a certain operation and ensure that it continues as desired. The surveillance process can be accomplished using a variety of different types of sensors. The data collected from the Arduino-based sensors will be compared with the target values (15). If the reading value of the sensor is found to be different from the target values, the control circuit will intervene to affect the allocated operation to keep it at the desired level of quality. As a result, the Smart food spoilage monitoring system focuses primarily on healthy food storage by surveilling and regulating a variety of parameters that affect food products. This control system employs storage units that are equipped with a variety of sensors that read the parameters influencing food quality (16).

### 1.3. Reasons for Food Spoilage

The recycling bins and the waste made are evidence of the food spoilage circumstances. The big reason for food spoilage is that a larger portion of food waste occurs in office eateries, small and large roadside eateries, group get-together events, and wedding receptions. These food surpluses are not only a widespread indicator of toxicity to the earth's atmosphere, but they also pose a slew of economic problems. According to current calculations, half of all of these foods are lost globally; the global amount of meal wastage is estimated as 1.3 billion tons, and it is projected to rise even more in the upcoming year, which is a concerning problem (17).

Food spoilage can be caused by a variety of human, chemical, and biological causes, including plants, enzymes found in plant food tissues, insects, parasites, and microbes (18). The most common and serious cause of food spoilage is microbial degradation. Food will invariably be polluted by such forms and amounts of microorganisms when microbes are commonly spread in nature.

**Figure 2:**
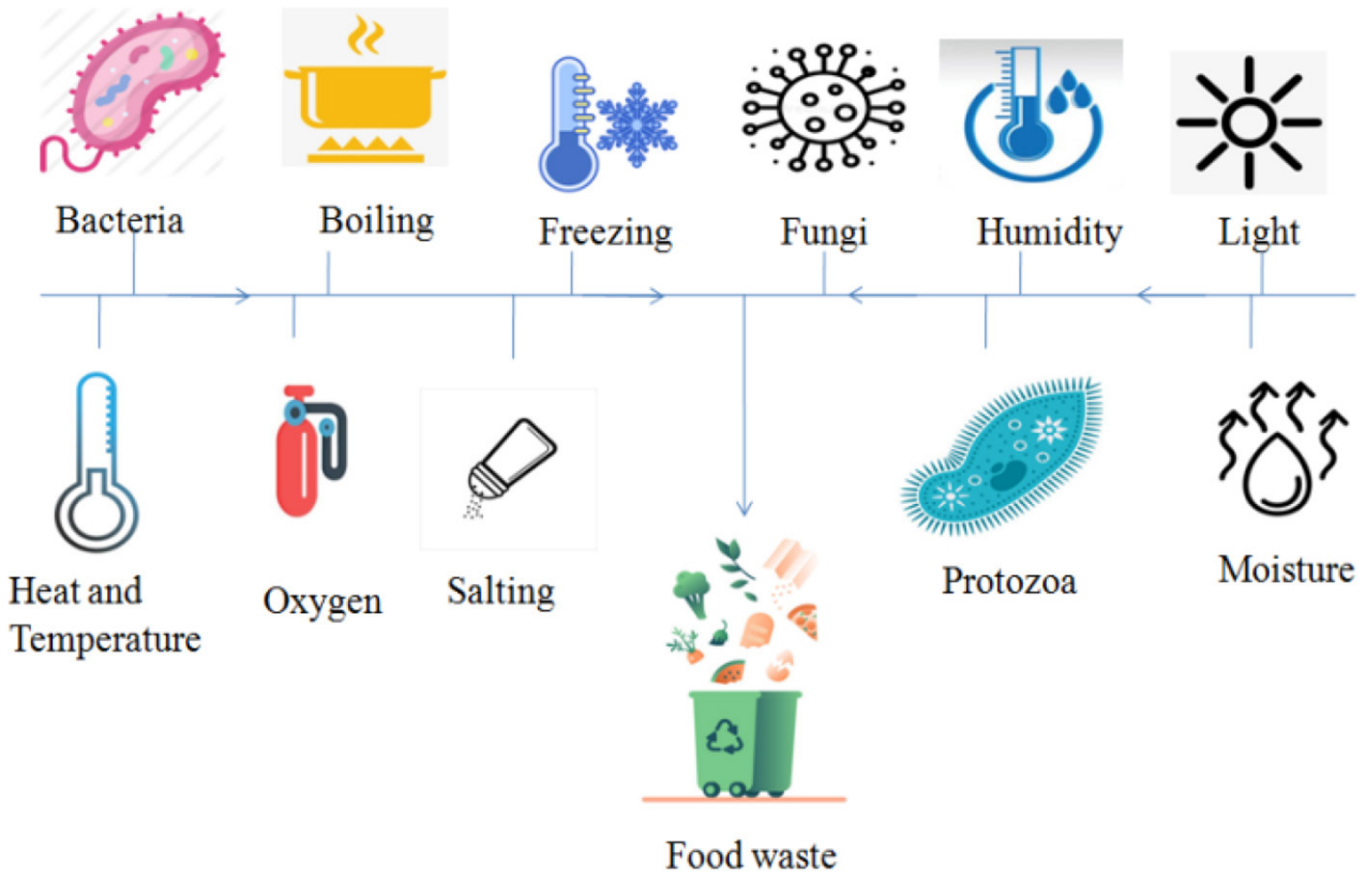
Factors of food spoilage.

Figure 2 shows the factors that can destroy fruits and veggies. In terms of heat, humidity, moisture, temperature, and oxygen content they have a threshold value. If the threshold value crosses then food spoiling starts. If we maintain a threshold value then we can vary the life of fruits and vegetables.

### 1.4. Motivation

The motivation behind Monitoring and analysis of food spoilage using Machine Learning is to keep track and manage food products in order to avoid spoilage caused by climatic and atmospheric changes. Food wastage concerns are focused on healthy eating habits and also concerns with the quality of food as there have been instances where poisonous chemicals have been discovered in fruits and veggies. Monitoring and analysis of food spoilage using Machine Learning saves time and provides accurate and consistent results.

### 1.5. Our Contribution

While reviewing the articles on food wastage, we have found that there are a few contributions to increasing the shelf life of food. To this end, we have found the research questions for this field. The major and novel contributions of this study are:

- Designing a smart system to keep track of the quality of food using gas, humidity, and temperature sensing mechanism.
- Designing an alert generation system to alert the user about the quality of food and the time spans of its deterioration.
- Development of a system to increase shelf life of food by maintaining the environment according to food.

## 2. Literature Review

Continuous sensing in a smart home is a unique sensor modality that has the ability to distinguish diverse everyday activities and indicate potentially dangerous situations for the person who lives there. One such situation is food rotting, which is the subject of this study (19). The authors have evaluated the smell signatures of two typical meals (milk and yogurt) that were kept at 25°C for a week using a metal-oxide sensor (MOS) based electronic nose. The highest absolute sensor responses were used to create feature vectors, and their components followed a smooth trajectory as the data aged. The two chemicals followed different trajectories during spoiling, according to principal component analysis (PCA).

Rajesh Megalingam et al. introduce a unique method for detecting food deterioration by combining picture classification with machine learning techniques and artificial intelligence (20). They have used AI, deep CNN networks, computer vision, and ML techniques such as the k clusters method for color classifications in pictures and its HSV values for spoiling detection to identify food rotting. The anaconda prompt is used to complete this project on the jupyter notebook platform. Also, Iwendi et al. employed an Artificial Intelligence approach for detecting and analyzing security levels on IoT using a network classifier. The study shows an impressive accuracy rate for the proposed application. In this view, we can say that AI is useful in almost every field nowadays.

Green et al. presented an electronic nose (e-nose) that consists of four gas sensors made of functionalized single-walled carbon nanotubes (f-SWNTs) and polymer nanocomposites, with the goal of becoming a simple monitoring device for microbiological spoilage and pollutants in canned food (21). The gas sensing signals were utilized as early indications of deterioration to assist in averting negative health impacts. In a static environment, the gas sensor array was tested to see how it responded to different volatile organic compounds (VOCs). Because they have an excellent sensing response to ammonia, which is one of the gases generated by microorganisms. These sensors are suitable for detecting microbial deterioration of canned food. The sample, which was canned tuna in mineral water, was opened and kept at room temperature (25°C). The odor linked with the deterioration of canned tuna was then monitored for 10 days using e-nose. PCA was also used to visualize the discrimination of microbial canned tuna spoilage status and analyze the smellprint of a specific level of ammonia contamination. PCA was found to be able to track changes in canned tuna deterioration, demonstrating its potential to be used for quality assurance of canned food in the daily life of a smart home resident.

When food is reversed in an inadvertent situation, such as an insufficient temperature, foodstuff rots as a result of the rapid breeding of food spoilage bacteria in warm and wet conditions where bacteria can easily be generated (22). The consequences can be even worse if humans eat those degenerative foodstuffs, as they may develop bromatoxism. The goal of this study is to design and simulate a wearable RFID patch for food spoilage monitoring with smart packaging that can be recognized and read temperature information by a device that supports near NFC technology *via* an attached circular antenna.

Everyone has a distinct opinion on whether or not a meal is rotten, which can lead to incorrect conclusions about the item's state (23). Foodborne sickness might result from a misinterpretation of the food condition. Due to this forecast ambiguity, there is still a desire for an electronic nose system to categorize if a meal is rotten. The goal of this study is to use an electronic nose to identify food deterioration in tomato-based Filipino cuisines. This study seeks to create a device with an array of sensors that can detect gases released by rotten tomato-based Filipino cuisines and to use an Artificial Neural Network as an algorithm to classify the sensor's data readings. The e-nose system's hardware includes a Gizduino 1281, a Raspberry Pi 3 Model B, a 20x4 LCD screen, seven MQ gas sensors, and one temperature/humidity sensor. The Artificial Neural Network data (24, 25) is trained using Stochastic Gradient Descent and the Back Propagation technique. The sensor chamber is positioned underneath the tomato-based Filipino food for simple detection of the gas sensors generated by the cuisine. The research might be beneficial in determining whether or not food has gone bad. This electronic nose device may detect the degree of rotting in a certain tomato-based Filipino dish. The researchers assigned rotting levels ranging from 0 to 12. Starting at 7:00 a.m. on Day 1, these levels correlate to the period when the meal is monitored every 4 h. As the trial progressed, the food deteriorated often between levels 5 and 6. The electronic nose system has a 3.85% mistake rate, according to the confusion matrix.

Benjamin et al. described a flexible UHF RFID sensor for detecting food quality (26). This sensor is based on inter-digital capacity found in an RFID antenna, onto which a coating of vegetal biopolymer has been placed. Electromagnetic coupling between the capacity and the biopolymer is, therefore, employed to vary the adaption coefficient between the chip and the antenna of the RFID tag based on food deterioration. Experimental measurements of an RFID-sensor exposed to a genuine food gas environment in the process of deterioration.

Food losses after harvest can range from 10 to 30%, owing to a variety of fungal and other microbes that degrade the food during transportation and storage (27). In addition to these direct losses due to spoiling, several bacteria generate natural poisons that make foods unsuitable for human ingestion. Other fruits and vegetables require waxes and water-loss barriers on the surface to retain quality and ideal sensory characteristics between farm and market. This study describes a study and development of an effective electrostatic spray application technology as well as a processing-line prototype designed particularly for food postharvest protection. The study provides experimental findings of comprehensive assessments of electrostatically applied protective sprays onto bananas for international transport, where both microbiological and mass-transfer data show usually 2.1–3.4-fold deposition benefits for food protection.

Microbial deterioration in milk is a major source of concern for those who want to eat healthily (28). The goal of this research is to use an electronic nose system (e-nose) with a variety of gas sensors as an initial tool for monitoring food deterioration in order to preserve food safety and human health. Nanoparticles gas sensors made of various polymers and f-SWCNTs are used in this device. When these gas sensors are exposed to the headspace of the milk, they respond to the VOCs present in the milk. Pasteurized milk was stored at 4 and 25°C for 9 days to monitor the development of spoiling. The odor pattern was examined using PCA to assess the capabilities of an electronic nose for freshness and milk spoiling detection. Monitoring the change in detecting responses revealed each sensor's capacity to identify the strength of the odor level. The findings of the discrimination demonstrate that the odor levels of samples maintained at 4°C do not change from the first to the ninth day, but the odor levels of samples held at ambient temperature increase with storage duration.

Food planning and quality assessments are becoming increasingly essential aspects of modern society (29). We may effectively analyze crop quality utilizing various learning approaches thanks to the scientific vision and agricultural advancements, which certainly replace inconsistent, unpredictable, and time-consuming human labor. The use of fuzzy logic to crop quality has effectively demonstrated its worth and established itself as a viable alternative to automation, indicating that it has a bright future ahead of it. This article examines some of the most important research findings on this subject.

Food safety is currently a key problem for the food business (30). Food businesses must develop quality monitoring systems in order to discover food quality issues early in the production process. Data manipulation and centralized storage, on the other hand, have become obstacles to dependability in a typical quality monitoring system. Furthermore, traditional quality monitoring techniques are typically ineffective due to a lack of effective automation. Fortunately, block-chain is a promising, tamper-proof, and decentralized technology. Furthermore, smart contracts, which are self-executing and self-verifying programs on the blockchain network, may execute transactions between mutually untrustworthy parties. This article offers an intelligent quality monitoring system for fruit juice manufacturing that combines smart contracts and quality rating algorithms. This system offers a high level of automation as well as a high level of dependability. Response surface models are created based on preproduction data in this system, and the best production condition for each step is determined. Smart contracts are used to capture production data on a blockchain during the actual production process.

Food is extremely essential in our day-to-day lives. The quality of food is deteriorating day by day as globalization progresses. To keep the food fresh, various food processing is done most of the time. Various preservatives or substances are added to the meal to give it a fresh or appealing appearance. The majority of food is now preserved using chemicals, resulting in food contamination. As a result of the pollution, numerous illnesses develop, prompting consumers to seek out healthier foods. Organic food is desired by the public in order to maintain a healthy lifestyle. So, in order to prevent difficulties linked with food that cannot be interpreted by humans, we need a gadget that can assist in determining the quality of food. There is a need for such a device that instructs us on how to eat sanitary meals. As a result, in order to meet this customer need, we created a gadget that determines if the food quality is good or terrible. This article examines the application of several sensors in the food business. Sensors such as pH sensors, gas sensors, and temperature sensors aid in determining the state of food. This system has a strong presence in restaurants, homes, and small businesses (31).

## 3. Existing Methods and Solutions

The goal of the smart meal tracking device is to track and regulate food products to avoid harm caused by atmospheric or climate variability. Food waste may also be caused by insufficient storage of foods. Through tracking and regulating different criteria concerning foodstuffs, the smart food monitoring system focuses on healthy food storage. This device employs storage units that have been implanted with a variety of electronic sensors that can interpret the parameters that affect foodstuffs.

### 3.1. Techniques for Food Preservation

**Figure 3:**
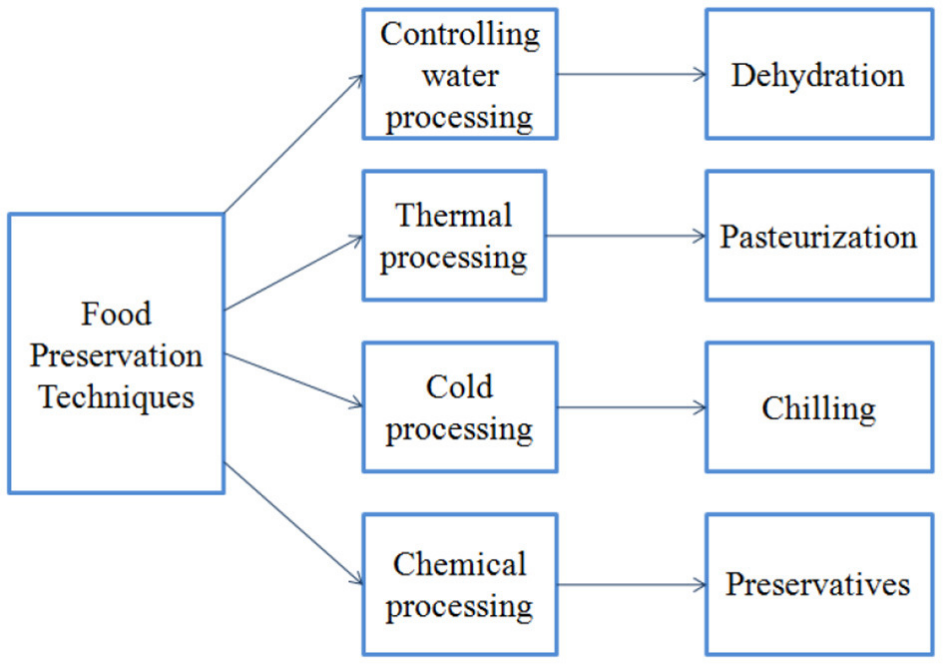
Food preservation techniques.

Food preservation's main goal is to save food from spoiling so it can be eaten. Gardeners also grow too much food at once, much more than can be consumed until it spoils. Food preservation also allows you to enjoy a wide range of foods all year long. The four techniques for food preservation are shown in Figure 3 (32).

### 3.2. Alternatives of Artificial Preservatives

Various natural preservatives derived from plants, insects, fungi, and alloy have antioxidant, antimicrobial, and anti-enzymatic effects in studies. Neem, basil, rosemary, and clove take out are encouraging alternatives for their synthetic equivalent (33).

Studies show that manufacturing companies are using chemical antioxidants which is the cause of hyperactivity in previously non-hyperactive people. Natural compounds derived from plants, organisms, or alloy may be useful substitutes. They may even be found as preservatives in meats, cosmetics, and pharmaceuticals, where they could be used as flavoring, binding, disintegrating, gelling, thickening, or suspension agents, even as automobiles.Researchers also used extracts from natural sources such as green tea, grape seed, and nisin, a bacteriocin known as a nutritious food preservative, to make chicken and turkey hot dogs in a series of trials (34). Table 1 represents the information regarding various food preservatives used for different kinds of fruits and vegetables. Table 2 represents the diseases caused using preservatives.

**Table 1:** Different types of food containing various kinds of preservatives.

**Preservatives**	**Food**
Sorbic acid	Syrups, sweets, dairy products, fruit products, fermented products, beverages
Tert butyl hydroquinone (TBHQ)	Fats, oils, snack foods
Tocopherols (vitamin E)	Oils
Ascorbic acid (vitamin C)	Fruit and acidic products
Butylated hydroxyanisole (BHA) and Butylated hydroxy -toluene (BHT)	Fats and oils, bakery products, cereals
Sodium sorbate	Mayonnaise, processed meats, dairy products, fermented products
Sodium and calcium propionate and Potassium propionate and propionic acid	Breads and other baked goods
Benzoic acid and sodium benzoate	Fruit products, margarine, and acidic foods
Calcium lactate	Olives, frozen desserts, jams, jellies, and dairy products
Calcium sorbate	Mayonnaise, dairy products, syrups, and margarine
Ethylene diamine tetra acetic acid (EDTA)	Dressings, canned veggies, and margarine
Methylparaben	Relishes, dressings, and beverages
Propylparaben	Cake, pastries, beverages, and relishes
Sodium nitrate and nitrite	Cured meats, fish, and poultry

**Table 2:** Dangerous food preservatives cause various diseases.

**Preservatives**	**Cancer possibility (Yes/No)**	**Asthma possibility (Yes/No)**	**Hypersensitivity possibility (Yes/No)**
Calcium/Potassium/Sodium propionate and propionic acid	No	Yes	Yes
Sodium and potassium nitrate	Yes	No	Yes
Sodium nitrite	Yes	Yes	Yes
Butylated hydroxyanisole (BHA)	Yes	Yes	Yes
Butylated hydroxytoluene (BHT)	Yes	Yes	Yes
Tert butyl hydroquinonesynthesiz-ed (TBHQ)	No	Yes	Yes
Sodium benzoate	Yes	Yes	Yes
Potassium and calcium sorbate and Sorbic acid	No	Yes	Yes
Benzoic acid	No	Yes	Yes
Propylparaben	No	Yes	No
Sulfur dioxide	No	Yes	Yes
Potassium bisulfite	No	Yes	Yes
Hexamethylen-etetramine	Yes	No	No
Sodium metabisulphite	No	Yes	No

### 3.3. Importance of Food Preservation

- Bacterial development and other forms of spoilage are inhibited by preservation processes, ensuring that the food is healthy enough to consume.
- Pickling competes with freezing, canning, and drying as a way to save produce from spoiling. Antioxidants, amino acids, and beneficial bacteria are commonly found in fermented foods.
- Food preservation delays the breakdown of rancid-causing fats while also inhibiting the development of microorganisms (such as yeasts) and other microorganisms.
- Feed storage expands the stock of food.
- Feed storage helps to reduce food waste. Excess foods that would have been discarded otherwise are processed and stored, adding to available stocks and reducing food waste (35).
- Dietary shortages can be reduced by storing food. Preserved foods help to bring diversity to the diet. In certain Middle East nations, there is a shortage of plantations due to arid environmental conditions. Importing new and dried fruits and veggies makes up for this shortcoming.

### 3.4. Reasons for Food Preservation

- The product could not be stored for a longer time as it could be spoiled.
- Using a new product that is in season and produced locally, remaking preserving jars, cutting food waste, and reducing food miles are all eco-friendly choices.
- Buy in bulk and seasonally to save costs.
- Artificial preservatives, BPA, and other artificial additives may not be used in home-prepared food.
- Convenience - in minutes, it is ready to eat.
- Both the canning process and sharing with families, colleagues, and neighbors need community participation. Teach the next generation how to do things.
- Have plans for unexpected events and/or injury.
- Personal fulfillment, we enjoy coming up with different flavor blends that you would not find in your grocery supermarket, as well as demonstrating how to use packaged foods in recipes.
- Learn new skills, such as how to use all of the fruit and how to cut down on food waste. Consider the following scenario: Apples - cut into slices (or use as pie filling) and squeeze the juice from the peels and cores.

## 4. Architecture

**Figure 4:**
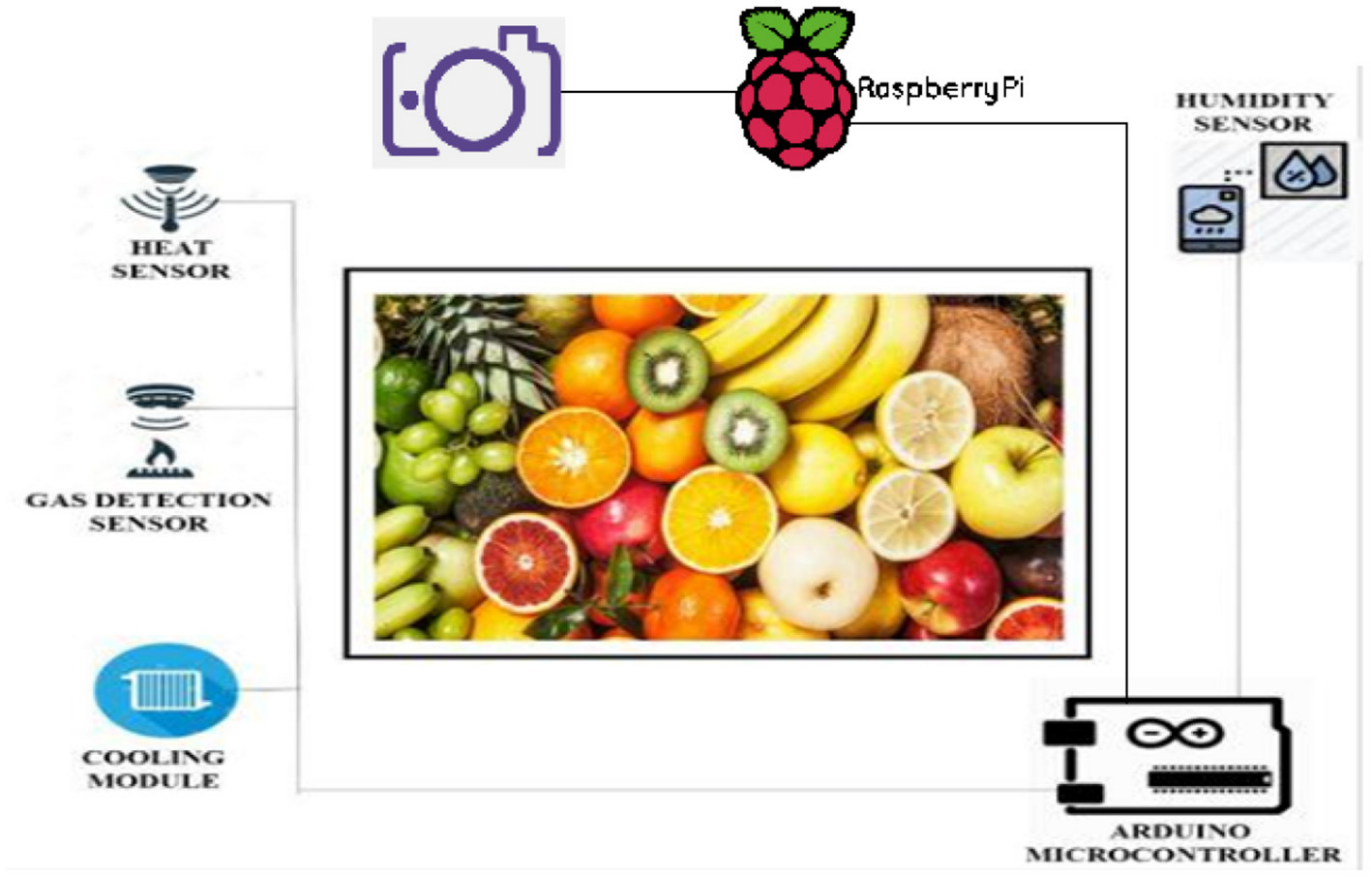
The architecture of Monitoring and analysis of food spoilage using Machine Learning.

The proposed solution includes new device architecture with few configuration modifications as per the requirement. The new device architecture includes a humidifier to maintain the humidity of the device and a cooling module that maintains the temperature for the food items. The prototype of the device is represented in Figure 4. Various electronic instruments have been used for monitoring. Furthermore, the registered values are used for monitoring purposes (36). The information gathered by various sensors can be correlated to the target values. The speed control kicks in and interprets the provided procedure to keep it running in the correct direction if the sensor readings do not match the key parameters. This theory can be used to create a method that can protect raw foods. This architecture consists of the following components:

- **Controller:** Arduino is used as a microcontroller.
- **Gas detection sensor:** A gas detector is a device that measures the number of gases in a given environment, and is often used as part of a protection system. Operators in the field where the leak is happening will be alerted by a gas detector (37, 38).
- **Humidifier:** A humidifier is a system that raises humidity (moisture) in a single room or a whole house. It is essentially an electrical appliance. Point-of-use humidifiers are widely used in the home to humidify a single room, and humidifiers are used in medical ventilators for greater patient convenience.
- **Heat sensor:** The key function of a heat sensor is to detect the heat that is present inside it. When the temperature around the heat sensor rises above its fixed point, it detects the heat and alerts us, allowing us to protect the devices from injury (39).
- **Humidity sensor:** A humidity sensor is a sensor that senses humidity and converts the information into an electrical current. Humidity sensors are available in a range of shapes and sizes, as well as with different features (40). The Humidity sensor first senses any humidity or moisture in the fruits or vegetables and it sends some value to the microcontroller if any moisture content is detected then an alert message sends to our mobile phone if the moisture content is not detected then again taking values from the microcontroller.
- **Cooling module (TEC1-12715-Thermoelectric Cooler 15A Peltier Module):** The heater, condenser, and fan device are all included in this element. A heater and a condenser are included in this module.
- **Light sensor:** Light sensors are electronic devices that monitor the intensity of natural or artificial light. Light energy is converted into an electrical signal by these devices. Light sensors are used in a variety of manufacturing application areas.

**Figure 5:**
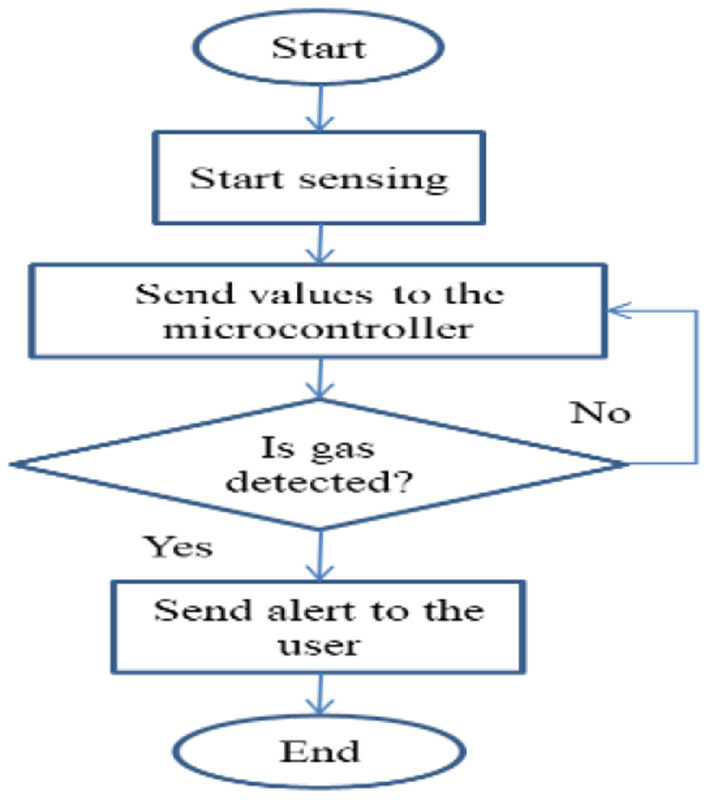
Gas detection sensor.

In Figure 5, the Gas detection sensor senses any gas or air coming from the fruits or vegetables, and it sends some value to the microcontroller if any gas content is detected then it alerts the user. If gas content is not detected then again values are taken from the microcontroller.

**Figure 6:**
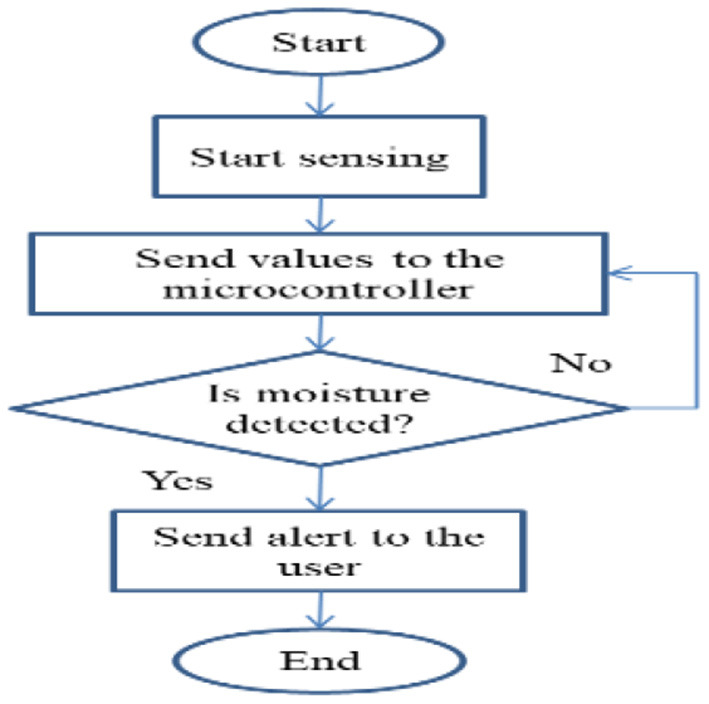
Humidity sensor.

**Figure 7:**
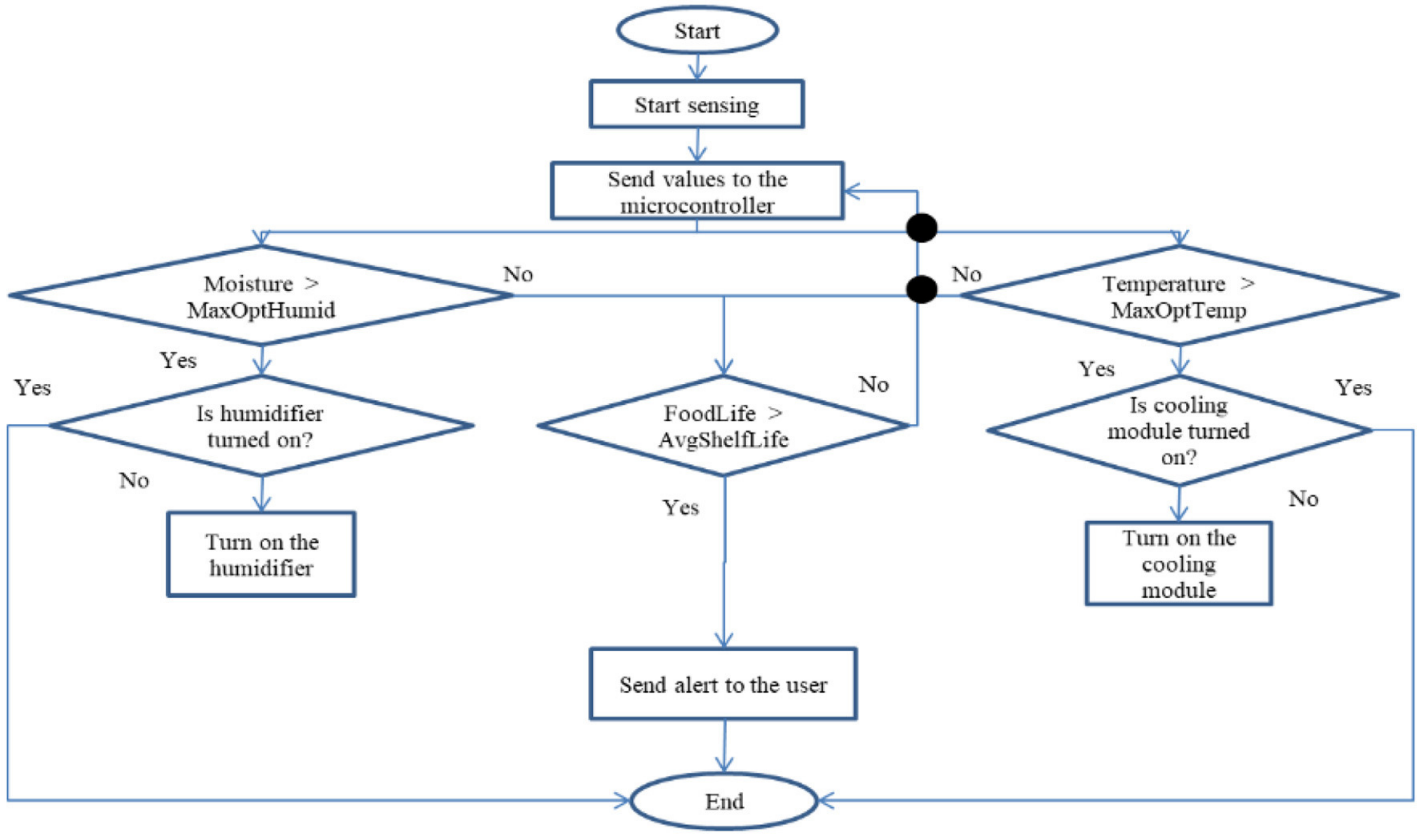
Heat sensor and cooling module.

In Figure 6, the Humidity sensor first senses any humidity or moisture in the fruits or vegetables, and it sends some value to the microcontroller. If any moisture content is detected then it alerts the user and if the moisture content is not detected then again values are read by the microcontroller. As shown in Figure 7, the heat sensor and cooling module senses any heat in the fruits or vegetables and it sends some value to the microcontroller. The complete process is given in Algorithm 1.

**Table:** **Algorithm 1** : Process (object).

1: Turn on the device
2: Capture the image of fruit or vegetable
3: Turn on the Cooling module
4: Turn on Humidifier
5: Store the optimal values of parameter according to captured object
6: Read the values of sensor for monitoring process of fruits or vegetables
7: if Gas content is detected **then**
8: go to step 20
9: else
10: go to step 21
11: end **if**
12: if Moisture content is detected AND moisture > maximum optimal humidity of object AND Humidifier is off **then**
13: Turn on the humidifier
14: else **if** heat content is detected AND temperature > maximum optimal storage temperature of object AND cooling module is off **then**
15: Turn on the cooling module
16: else **if** life of object > average shelf life of object **then**
17: go to step 20
18: else
19: go to step 21
20: end **if**
21: Send alert to the user
22: Capture an image of fruit or vegetable
23: go to step 7

## 5. Working Principle

We have different types of sensors that play a major role in the detection and monitoring of food spoilage. It includes the camera sensor, humidity sensor, gas sensor, and heat sensor. The camera sensor captures the image of fruit or vegetable. The humidity sensor senses the humidity of the environment. If it is below the threshold value then the humidifier increases the humidity up to the threshold value. The temperature sensor monitors the temperature for the predefined threshold value which is controlled by Arduino. Arduino is directly connected to the Raspberry pi, which acts as a mini computer having own processing unit and memory. Whenever temperature reaches above the value of the threshold, the cooling module gets turned on. The gas sensor detects early spoilage *via* detecting a little amount of gas emission of the food items. It sends the value to the Arduino, after receiving the values, Arduino pushes the values to the cloud. The web application notifies the user *via* a buzzer or voice-activated commands or through the display messages.

## 6. Smart Food Life Prediction

The food life cycle has different stages which include the following stages: Fresh Food; Food spoilage just started; Spoilage Food. For prediction, we have trained 50 types of fruits and vegetables over different three classes. We proposed a CNN for object detection and prediction model. This is trained over three different classes.

Object detection is a difficult task in the realm of computer vision. Machine learning models when trained on a large enough number of photos can be used to detect and distinguish objects. We will identify the fruit or vegetable by using the Convolutional Neural Network (CNN) to calculate the threshold values for different parameters e.g., gas, temperature, and heat as these values are different for a different type of food. The networks will be trained in a supervised way, with photos of the fruits serving as input and tags serving as a result. The CNN model will be able to correctly anticipate the fruit label after successful training.

We have utilized the deep learning approach to train our model on datasets of fruits and vegetables. We used the Fruits360 dataset, which is openly available and may be downloaded from GitHub or found on Kaggle (41). It has around 90,380 pictures in various categories. This dataset contains high-resolution pictures, which are required for a successful classifier.

In our model, there are a total of 11 layers. The output layer is SoftMax, and there are four convolutional layers, four max pooling layers, and two fully connected layers. The input layer is a convolutional layer with a 16, 5 x 5 x 4 filter and 2 x 2 max-pooling with stride = 2. Convolutional layer 4 has a parameter of (5 x 5 x 64) and a dimension of 128. The model has been trained on 50 different types of fruits and vegetables, and it is also capable of identifying multiclass images.

## 7. Results and Discussion

Information gathering is an important and time-consuming task. Regardless of the field of study, precise data collection is crucial to maintaining cohesion. Food spoilage is a very concerning topic nowadays because this time is needed to have a strong immune system, and there is no existing solution that helps to prevent spoilage to some extent. The Fruit360 is the data set that was used in this study. We have to gather a dataset from the Kaggle dataset website. The dataset contains images of different fruits and vegetables. We have used this dataset for CNN model training and object detection.

Fruits and Veggies—We have created a dataset after careful research. The dataset contains 50 types of fruits and vegetables with their optimal storage temperature range, optimal humidity range, approximate storage life range, and freezing point are included in this collection (42). Dataset is used for spoilage alert generation messages in mobile. Table 3 describes the dataset attribute with its value, type, and description. All the value of each column represents numeric and floating point values except the first column.

**Table 3:** Description of the fruits and veggies dataset.

**Columns**	**Description**	**Value**	**Type**
Names of fruits and vegetables	Different types of fruits and vegetables	NA	String
Minimum optimal storage temperature	Minimum temperature in which fruit or vegetable remain fresh	Multiple minimum optimal temperature values	Numeric
Maximum optimal storage temperature	Maximum temperature in which fruit or vegetable remain fresh	Multiple maximum optimal temperature values	Numeric
Freezing point	This cooling point in which fruit or vegetable remain fresh	Multiple freezing point values	Numeric
Minimum optimal humidity	Minimum humidity in which fruit or vegetable remain fresh	Multiple minimum optimal humidity values	Numeric
Maximum optimal humidity	Maximum humidity in which fruit or vegetable remain fresh	Multiple maximum optimal humidity values	Numeric
Minimum approximate storage life	At least number of days in which fruit or vegetable remain fresh	Multiple minimum approximate storage life values	Numeric
Maximum approximate storage life	At most number of days in which fruit or vegetable remain fresh	Multiple maximum approximate storage life values	Numeric
Average shelf life	Average of minimum (start spoiling) spoilage time and maximum (after spoiled) spoilage time	Multiple average shelf life values	Numeric

**Figure 8:**
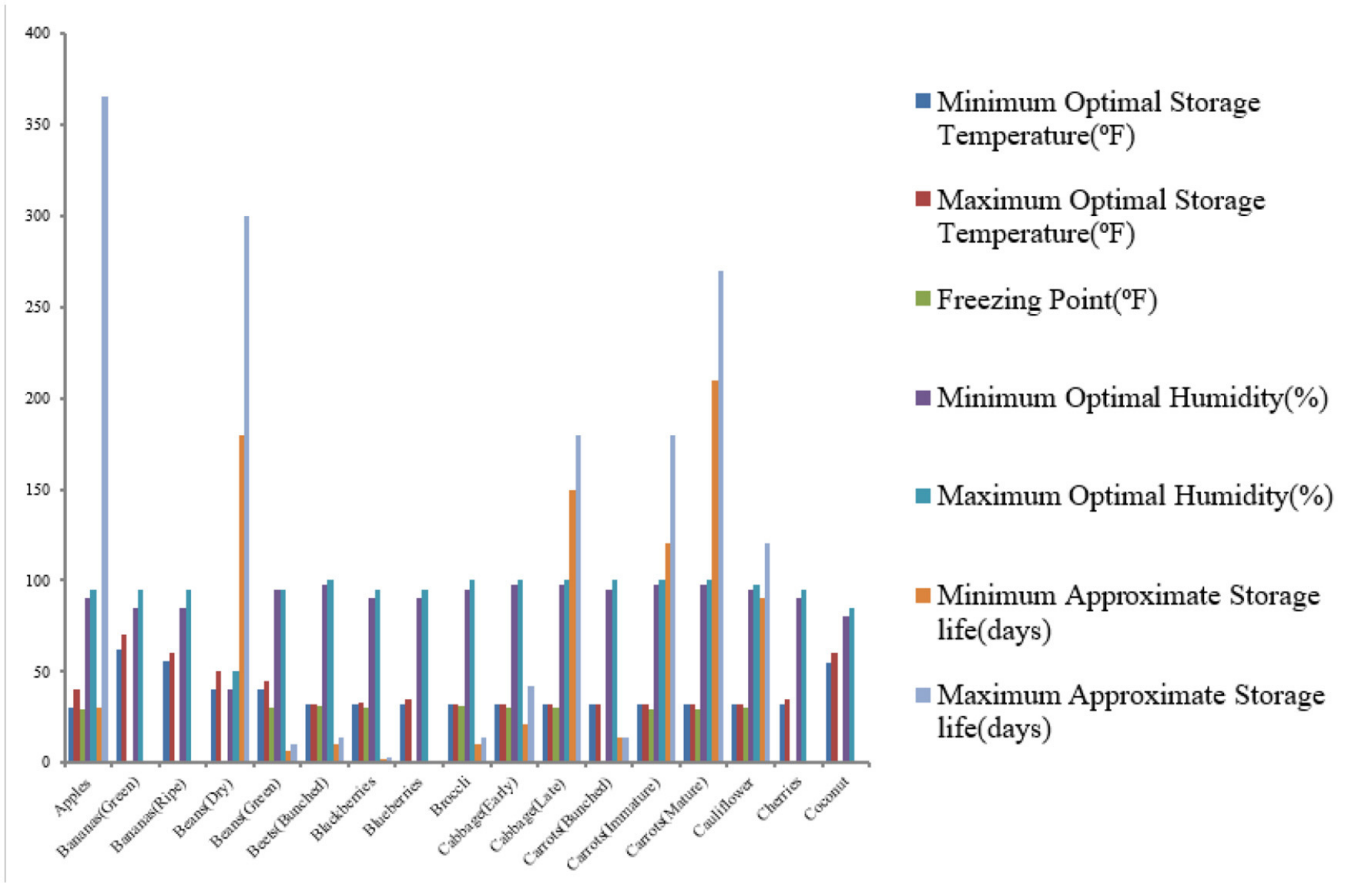
Comparison among different fruits and vegetables with respect to their various attributes.

Figure 8 represents the relation between attributes like minimum optimal storage temperature, maximum optimal storage temperature, Freezing Point, minimum optimal humidity, maximum optimal humidity, minimum approximate storage life, and maximum approximate storage life of 17 fruits and vegetables. We have selected 15 fruits or vegetables out of 50 fruits or vegetables for our experimental purpose. This figure represents the comparison among standard parameter settings of different food items. Then we put the food item in the proposed device, which further analyzes the item and sets the parameter values accordingly. Finally, the proposed approach is used to monitor the item and maintain the environment accordingly. In the end, we monitored the final shelf life of the food item. In this experiment, we try to extend the maximum approximate storage life of fruits and vegetables, which is represented in Table 4.

**Table 4:** Experimental analysis of fruits and vegetables.

**Name of fruits or vegetables**	**Minimum temperature (**°**F)**	**Maximum temperature (**°**F)**	**Average shelf life (days)**	**Maximum approximate storage life (days)**	**After experimental analysis (days)**
Broccoli	32	32	11	14	16
Cabbage (Early)	32	32	41	42	44
Carrots (Immature)	32	32	35	180	181
Cauliflower	32	32	14	120	122
Cherries	30	31	6	14	15
Grapes	31	32	6	56	55
Kohlrabi	32	32	7	90	91
Gooseberries	31	32	3	28	29
Leeks	32	32	11	90	91
Parsley	32	32	6	90	91
Plums	31	32	4	35	36
Eggplant	46	54	2	7	9
Blackberries	32	33	6	3	4
Corn (Sweet)	32	32	7	8	9
Cucumbers	50	55	11	14	15

The average shelf life of any fruit or vegetable is a time period in which fruits or vegetables are healthy for eating. After this period of time, it is harmful for health to consume this fruit or vegetable as spoilage may have taken place previously before the average shelf life. Every fruits or vegetables have a different minimum optimal storage temperature below which they started spoiling. So, careful minimum optimal storage temperature must be maintained.

**Figure 9:**
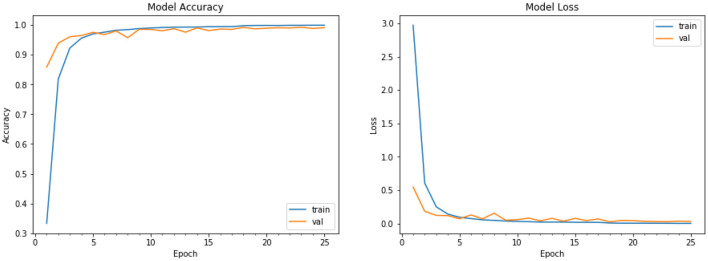
Performance graph of Training Loss vs. Validation Loss.

**Figure 10:**
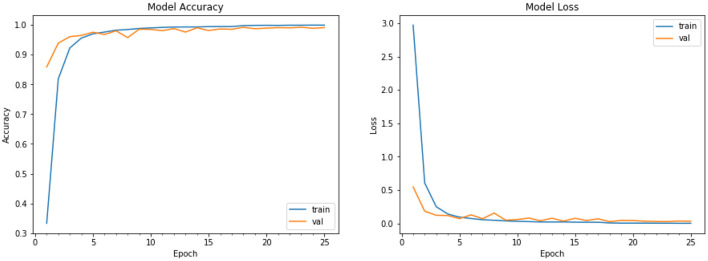
Performance graph of training accuracy vs. validation accuracy.

The performance graph for our model is plotted for both the training and validation data. We show the training data loss vs. validation data loss graph, as well as the training data accuracy and validation data accuracy of the CNN model for fruits and vegetables, for object detection. The performance graph plot shows the improvement in accuracy as the number of epochs increases, as well as whether or not our model has been correctly trained. The CNN model's training data loss vs. validation data loss, as well as training data accuracy and validation data accuracy, are shown on the graph shown in Figures 9, 10. There are three different fit situations for the model: over fitting, under fitting, or perfect fit.

- **Over fitting:** When the Training loss is considerably less than the Validation loss, this scenario occurs.
- **Under fitting:** This situation occurs when the Training loss exceeds the Validation loss, i.e., Training loss > Validation loss.
- **Perfect fit:** This circumstance occurs when Training and Validation losses are approximately equal or converging over time, indicating that we are doing things correctly.

As seen in our graph of Figure 9, it is virtually converging to indicate that our model is free of over fitting and under fitting. The plot of training and validation accuracy after each epoch is shown in Figure 10, our model is virtually ideal in this case since the validation and training accuracy are nearly identical. So, our model has a 95% accuracy rate, which is excellent enough to recognize the kind of vegetable and fruit from a picture. It also works well for single items, such as single fruits or vegetables. It also has the ability to forecast the class of numerous objects. For 50 different sorts of categories, the approach works perfectly. It provides extremely precise results for the photos that are fed into the model.

## 8. Conclusion and Future Scope

This study presents a novel technique for Monitoring and analysis of food spoilage using a sensor bases system. The device proposed in this study is able to preserve food for more days. Additionally, food items can be prevented from getting spoiled by increasing their lifespan. It monitors the quality of food items and keeps notifying the user with voice-activated commands or *via* display, and it also generates alerts to the user with the predicted remaining time of the food spoilage. The proposed device shows an accuracy of 95%.

The proposed smart device can be improved by applying image processing and machine learning algorithms to detect early spoilage. This can be utilized in refrigeration systems for detecting food items, spoilage and monitoring for prevention of food spoilage. The device can be incorporated into food transportation containers which would allow tracking and detecting the spoilage if any during transportation. The device could also be tested for different variety of foods as well.

## Data Availability Statement

The datasets presented in this study can be found in online repositories. The names of the repository/repositories and accession number(s) can be found below: https://docs.google.com/spreadsheets/d/1Cz8N7z6tJ8FouYnbbn5shy2D71Ln5P0W/edit?usp=sharing&ouid=105439467837665352046&rtpof=true&sd=true.

## Author Contributions

UB and ES carried out the experiment. ES wrote the manuscript with support from UB. ES fabricated the device with the help from RA, AB, and MH. UB supervised the project. All authors contributed to the article and approved the submitted version.

## Conflict of Interest

The authors declare that the research was conducted in the absence of any commercial or financial relationships that could be construed as a potential conflict of interest.

## Publisher's Note

All claims expressed in this article are solely those of the authors and do not necessarily represent those of their affiliated organizations, or those of the publisher, the editors and the reviewers. Any product that may be evaluated in this article, or claim that may be made by its manufacturer, is not guaranteed or endorsed by the publisher.
